# Supplementary material for: 3D printed sub-terahertz photonic crystal for wireless passive biosensing
Source: Commun Eng. 2024 May 17;3:69. doi: 10.1038/s44172-024-00213-4 (PMC11101475; doi:10.1038/s44172-024-00213-4)
Supplement: Supplementary file 2 — Supplementary Information [file 44172_2024_213_MOESM2_ESM.pdf]

Supplementary Information

Supplementary Notes

Note 1: FOM

The effect of the analyte on the frequency shift Δf_{r} can be expressed using the perturbation theory with the approximation that the volume of the analyte is much smaller than the sensor[1]:

$$\frac{\Delta f_{\mathrm{r}}}{f_{\mathrm{r}}} \approx -\frac{\Delta n}{n} \cdot \frac{\iiint V_{\mathrm{a}} dV \varepsilon |\vec{E}|^2}{\iiint V_0 dV \varepsilon |\vec{E}|^2}, \qquad (1)$$

Where V_{a} is the volume of the resonator and V_0 is the volume of the whole area including the resonator, the analyte, and the surrounding air. The fractional frequency change in frequency is related to the fraction of the electric field energy interacting with the analyte. A resonator sensor with a high field concentration can overcome the difficulty that the thickness of the pathogen layer is very small compared to the wavelength of electromagnetic wave. The sensing performance of a resonator sensor detecting thin-film analyte can be evaluated using a figure-of-merit (FOM) parameter regarding the unit thickness of the analyte[2]:

$$\mathrm{FOM} = \Delta f_{\mathrm{r}} / (\mathrm{FWHM} \cdot \Delta n \cdot h_{\mathrm{a}}), \qquad (2)$$

where FWHM is the full width at half maximum and also called 3 dB bandwidth and h_{a} is the thickness of the analyte. The FOM considers not only the frequency shift, but also the amplitude variation, which should be higher than noise for a unambiguous detection. The Q-factor can be calculated by using the equation[2]:

$$Q = f_{\mathrm{r}} / \mathrm{FWHM}. \qquad (3)$$

Thus, FOM can be rewritten as:

$$\mathrm{FOM} \approx \frac{Q}{n \cdot h_{\mathrm{a}}} \cdot \frac{\iiint V_{\mathrm{a}} dV \varepsilon |\vec{E}|^2}{\iiint V_0 dV \varepsilon |\vec{E}|^2}. \qquad (4)$$

It can be seen that a high Q-factor and a high field concentration can enhance the sensing capacity.

Note 2: Radar equation

The wireless sensor works similarly to an RFID tag. The maximum reading range achieved by a wireless sensor can be calculated using the radar equation[3]:

$$R_{\mathrm{max}} = \sqrt[4]{\frac{P_{\mathrm{T}} G^2 \lambda^2 \sigma_{\mathrm{sensor}}}{(4\pi)^3 P_{\mathrm{R}}}}, \qquad (5)$$

where P_{T} and P_{R} represent the transmitted power and receiver sensitivity of the reader, respectively. G denotes the reader antenna gain assuming a mono-static radar setup (i.e., the same antenna is used for transmitting and receiving) and σ_{sensor} is the radar cross section (RCS) of the sensor measured at the resonance frequency. The equation indicates that the maximum detection range depends on the RCS of the sensor for a given reader system. Further, the maximum detection range scales with the wavelength. This limits the wireless reading capabilities of devices operating at high frequencies, i.e., optical photonic crystals.

Note 3: Measurement setup

Figure S1 shows the wireless measurement setup used to characterize the sensor. On the left-hand side a 26 dB horn antenna is attached to the rectangular waveguide flange for transmitting and receiving electromagnetic waves. On the right-hand side the sensor is mounted a 3D printed holder made of Acrylonitrile Butadiene Styrene.

The dielectric rod antenna (DRA) of the sensor is aligned to the center of the horn antenna using a laser aligner, which produces horizontal and vertical green light lines as shown in Figure S1. The distance between the tip of the DRA and the edge of the horn antenna is defined as L. By varying L, the reading range of the sensor can be studied.

Note 4: Prepared solution

Figure S2 shows the prepared bovine serum albumin (BSA) solution at the concentrations of 2.4, 4.8, 7.2, and 9.6 g L^{-1}, respectively. As the concentration increases, more combined protein is suspended due to the saturation of the solution. To achieve a relatively uniform dispersion, the bottle is shaken before each measurement.

Note 5: Effect of the resonant frequency on the resonant shift and FOM

The resonance shift due to the analyte depends on the resonant frequency. To study this, the resonator with two ports is scaled to different frequencies ranging from 100 GHz to 1000 GHz and simulated in CST. A thin film of analyte with a constant refractive index (1.8) and a constant thickness (0.1 μm) is deposited on the walls of the slot in the resonator. Their resonance shifts and FOMs are simulated and plotted in Figure S3. The results indicate that as the resonant frequency increase, the resonance shift increases greatly and the sensitivity is enhanced.

Note 6: Effect of the analyte covering different areas on the sensor

In this manuscript, the analyte is deposited on the walls of the slot as a proof of principle. However, it is important to note that a larger covered area by the analyte leads to stronger interaction between electromagnetic wave and the analyte. As a result, the resonant shift due to the analyte is increased and the sensitivity is improved. To investigate the effect of different positions, the resonator is simulated with the analyte positioned on different areas in CST. The different positions of the analyte and the simulated resonant shift due to it are presented in Figure S4-S9.

Note 7: More details of simulation setup

To optimize the sensor and analyze its sensitivity, the transmission parameter of the resonator with two tapers is simulated with CST as shown in Figure S10. Both tapers are inserted into a WR10 rectangular waveguide (shown in yellow transparent) for excitation and receiving. Waveguide ports are used for the WR10 waveguide. Because the expected frequency spectrum has a resonance peak with high Q factor, a frequency domain solver is chosen to reduce simulation time. When using a time domain solver, the simulating time of a high-Q resonator is very long and the simulated Q factor depends on the simulation time. Since the radiation pattern of the resonator is not needed, an open background is used. In the solver setup, a single frequency sample is set near the resonant frequency and automatic frequency samples are chosen. Furthermore, adaptive mesh refinement is activated to improve accuracy. The material of the resonator is defined as: Epsilon=9 and Tangent delta=0.00022. The dimensional parameters such as radius of holes, slot width, and shift of the holes are studied by a parameter sweep.

Supplementary Figures

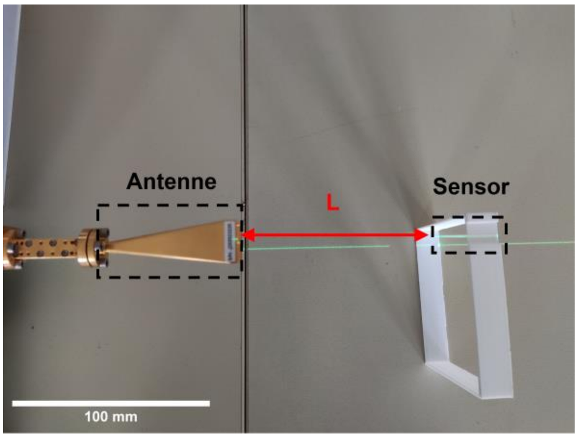

Figure S1. Setup of wireless Measurement.

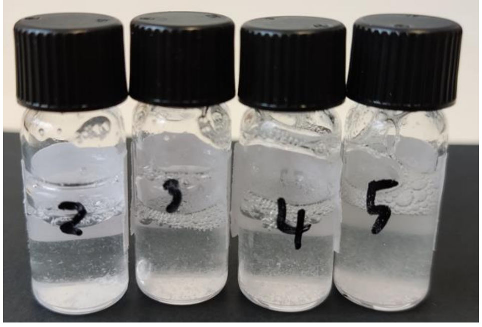

Figure S2. BSA in destilled water, at the concentrations of 2.4, 4.8, 7.2, and 9.6 g L–1, respectively.

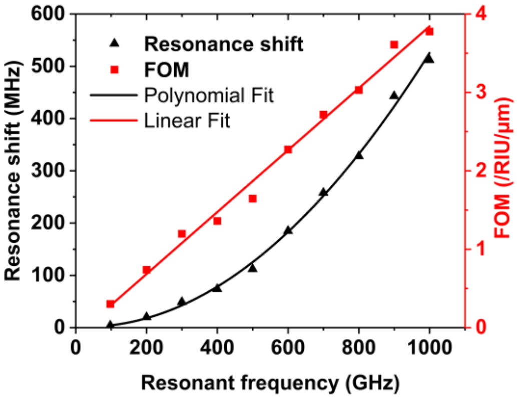

Figure S3. Resonant shift of the resonators with different resonant frequencies with a 0.1 µm thin film as the analyte.

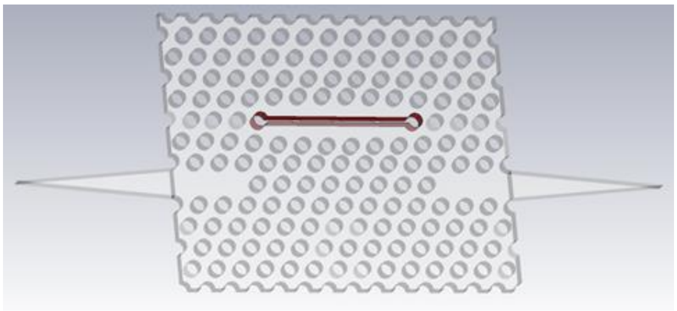

Figure S4. The analyte (in red) covers the wall of the slot (area 1), which is the same as in the manuscript. The simulated resonant shift is 24 MHz.

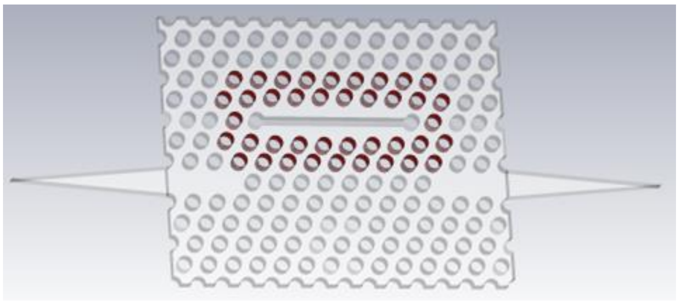

Figure S5. The analyte (in red) covers the wall of the holes around the slot (area 2). The simulated resonant shift is 14 MHz.

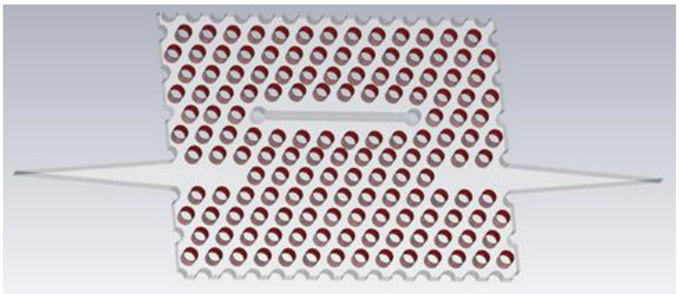

Figure S6. The analyte (in red) covers the wall of the holes of the whole sensor (area 3). The simulated resonant shift is 18 MHz.

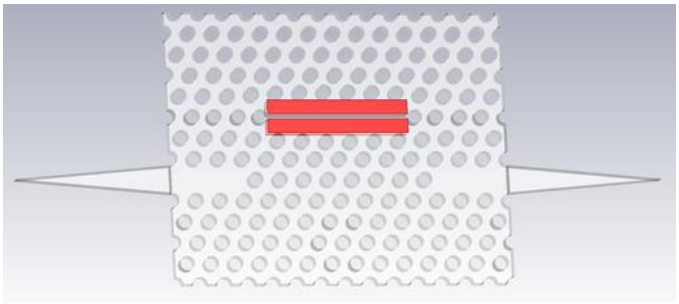

Figure S7. The analyte (in red) covers the top and bottom surface around the slot (area 4). The simulated resonant shift is 10 MHz.

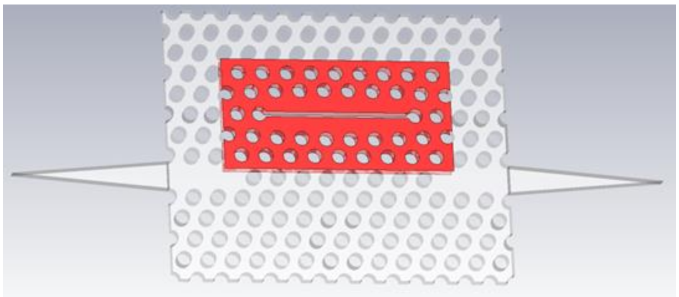

Figure S8. The analyte (in red) covers the top and bottom surface in a large area of the resonator (area 5). The simulated resonant shift is 14 MHz.

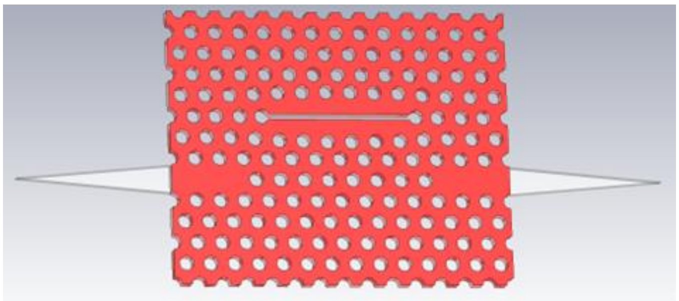

Figure S9. The analyte (in red) covers the top and bottom surface of the whole resonator (area 6). The simulated resonant shift is 14 MHz.

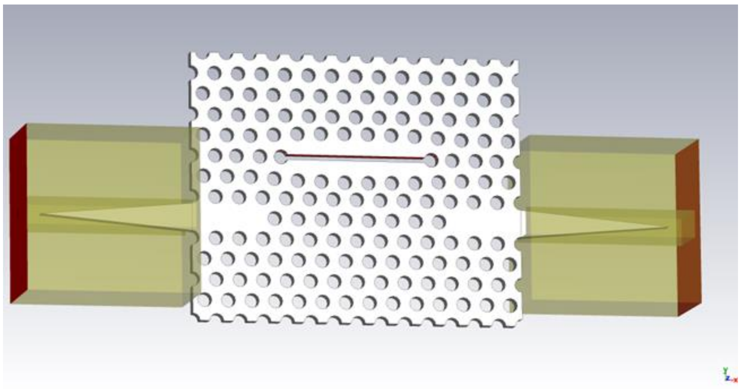

Figure S10. The model of the resonator and the simulation setup in CST.

Supplementary References

1. Zhao, Y. *et al.* Sensitive and Robust Millimeter-Wave/Terahertz Photonic Crystal Chip for Biosensing Applications. *IEEE Access* **10**, 92237–92248 (2022).
2. Beruete, M., Jáuregui-López, I. & Jáuregui-López, I. Terahertz Sensing Based on Metasurfaces. *Adv. Opt. Mater.* **8**, 1–26 (2020).
3. Borgese, M., Genovesi, S., Manara, G. & Costa, F. Radar Cross Section of Chipless RFID Tags and BER Performance. *IEEE Trans. Antennas Propag.* **69**, 2877–2886 (2021).
